# Heart Disease and Sea Winds

**Published:** 1871-01

**Authors:** 


Heart Disease and Sea Winds.—In a lecture by Alfred Haviland, on the Geographical Distribution of Diseases in England and Wales, the lecturer stated as the result of careful investigation, that whenever the prevailing sea winds have uninterrupted access, there is a low mortality from diseases of the heart, and that the opposite condition occurs where the atmospheric tidal wave has not free access. The law may hold in England, but it certainly does not apply to San Francisco.

